# Association Between Lycopene and Metabolic Disease Risk and Mortality: Systematic Review and Meta-Analysis

**DOI:** 10.3390/life15060944

**Published:** 2025-06-12

**Authors:** Isabel Viña, Alicia Robles, Juan R. Viña

**Affiliations:** 1 IVB Wellness Lab, C/Colón 12, 46004 Valencia, Spain; alicia.robles@ivbwellness.com; 2 Departamento de Bioquímica y Biología Molecular, Facultad de Medicina, Instituto INCLIVA, Universitat de València, 46010 Valencia, Spain; juan.r.vina@uv.es

**Keywords:** lycopene, metabolic syndrome, non-alcoholic fatty liver disease, insulin resistance, *diabetes mellitus*

## Abstract

Background: Lycopene, a dietary carotenoid with antioxidant properties, protects against sun-induced skin damage, prostatic conditions such as chronic prostatitis, and cancer; however, its role in metabolic disorders, including metabolic syndrome and nonalcoholic fatty liver disease (MAFLD), remains unclear. This study aimed to systematically assess the association between lycopene levels (serum and dietary) and the risk of metabolic diseases. Methods: This study adhered to the PRISMA guidelines and was prospectively registered on the Open Science Framework (OSF). We searched PubMed, Scopus, Web of Science, and Medline via WoS. Pooled odds ratios (OR), hazard ratios (HR), and mean differences (MD) with 95% confidence intervals (CIs) were calculated using fixed or random-effects models based on heterogeneity. Results: Twenty-nine studies were included, of which twenty-five were eligible for the meta-analysis. Pooled analysis showed that the individuals with the lowest serum lycopene levels had a significantly higher risk of MAFLD (OR = 1.39, 95%CI: 1.02–1.89, *p* = 0.0388). No significant associations were found between HbA1c levels, diabetes history, and weight status. Although not statistically significant, a clear trend of patients with DM having lower lycopene levels than the control group was observed (MD = −0.09, 95% CI: −0.19 to 0.00, *p* = 0.054). Comparisons based on weight status showed no significant differences. Conclusions: While lower serum lycopene levels are significantly associated with increased MAFLD risk, their impact on glycemic control remains inconclusive, underscoring the need for targeted clinical research.

## 1. Introduction

The development of metabolic disorders, such as impaired glucose tolerance, hypertension, and hyperlipidemia, is primarily driven by excessive oxidative stress and chronic low-grade inflammation [1]. Both processes play a central role in the initiation, progression, and perpetuation of various alterations in biochemical and molecular pathways that lead to the onset of metabolic dysfunction, thereby increasing the risk of developing or worsening a wide range of diseases (from cardiometabolic conditions to autoimmune disorders as well as neurological and neurodegenerative diseases). In particular, persistent low-grade inflammation promotes the onset of metabolic diseases, making it a critical therapeutic target, as demonstrated in numerous clinical trials involving statins, in which part of their beneficial effect on mortality reduction is directly attributed to their anti-inflammatory effects [1]. Thus, identifying effective nutritional strategies is a promising approach for mitigating the burden of these widespread conditions [2].

In this context, it is essential to understand how oxidative stress and chronic low-grade inflammation interact in a mutually reinforcing manner, contributing to the progressive deterioration of metabolic health. Oxidative stress not only initiates inflammation but also sustains it by increasing the production of oxidizing free radicals that damage various cellular structures, including the endothelium. This, in turn, activates the immune system’s defense mechanisms, which, by their nature, further amplify inflammation. Under homeostatic conditions, this response is self-limiting and mediated. However, when the intensity and duration of oxidative stress and inflammation exceed the body’s antioxidant capacity, a vicious cycle is established. This leads to damage to biochemical pathways and cascades, ultimately predisposing individuals to the development of various metabolic diseases [3].

Given the central role of oxidative stress and inflammation in the pathogenesis of metabolic dysfunction associated with obesity, metabolic syndrome, and various other diseases, considerable attention has been directed toward the potential protective role of natural antioxidants, particularly carotenoids. Adipose tissue and the liver are the primary sites of carotenoid storage [4]. Due to their lipophilic nature, once absorbed, carotenoids diffuse across plasma membranes and accumulate within cells, where they exert, among other effects, antioxidant activity through multiple mechanisms. Among the various tissues where carotenoids are stored, adipose tissue is one of the most receptive to lycopene incorporation, thereby contributing to the complex metabolic processes occurring in both adipose tissue and serum [5,6]. Notably, obesity is associated with reduced plasma carotenoid concentrations and carotenoid deficiencies, likely due to increased utilization and/or impaired bioavailability or function [7].

Lycopene is one of the most prevalent carotenoids in human tissues [8]. Since the human body cannot synthesize lycopene, it must be obtained through diet [9]. Tomatoes, especially tomato-based concentrates, are the primary dietary sources of lycopene [10]. Most lycopene in the body is derived from natural food sources [11]. Its bioavailability depends on mechanical processes, such as chewing and gastrointestinal peristalsis, which aid in the release of lycopene from the food matrix. In the stomach, digestive enzymes, gastric acid, and mechanical churning further facilitate lycopene liberation [12].

Once in the small intestine, lycopene is incorporated into lipid micelles through the aid of bile acids and digestive enzymes [12]. Lycopene absorption is influenced by various biological and lifestyle factors, including age, sex, plasma lipid levels, hormonal status, body composition, diet composition, and habits such as smoking and alcohol consumption [13]. As a lipophilic molecule, lycopene absorption is enhanced by dietary fat but inhibited by the presence of dietary fiber and competing carotenoids, such as β-carotene [5]. After absorption, lycopene is primarily distributed to the liver, prostate, and adrenal glands, with smaller amounts deposited in the brain, skin, and adipose tissue [4,11]. In the liver, lycopene undergoes oxidative and enzymatic metabolism to form bioactive metabolites, including apo-lycopenals and apo-lycopenones [14].

While oxidative stress and chronic inflammation are well-established contributors to the development of metabolic diseases, such as MAFLD, obesity, and type 2 *diabetes mellitus* [15], the role of lycopene remains inadequately defined. Although observational studies have suggested that lower circulating lycopene levels are associated with an increased risk of metabolic disorders, the evidence remains fragmented owing to inconsistencies in study design, population characteristics, supplementation protocols, and outcome measures [16]. Moreover, the potential mechanisms through which lycopene may exert protective effects—such as the modulation of insulin-like growth factor-1 (IGF-1), a key factor in explaining the proposed protective effects of lycopene with respect to cancer risk [17] as well as its interaction with oxidative stress pathways—are not yet fully elucidated [18].

Despite the growing interest in the potential metabolic benefits of lycopene, the current evidence remains fragmented and inconclusive. Existing studies vary widely in terms of lycopene sources (dietary vs. supplemental), the populations studied, and the metabolic outcomes assessed [19]. Moreover, while some reviews have examined the effects of lycopene on specific conditions (e.g., obesity or type 2 diabetes), a comprehensive synthesis of its association with a broader spectrum of metabolic dysfunctions, including MAFLD, insulin resistance, and IGF-1-related pathways, is lacking [20]. This limits our understanding of the preventive or therapeutic role of lycopene in interconnected metabolic outcomes.

This combined systematic review and meta-analysis is the first to evaluate the association between serum lycopene levels and the risk of MAFLD; assess the effects of lycopene supplementation on metabolic biomarkers, including IGF-1; and synthesize the available evidence to better reveal lycopene’s potential role in the prevention and management of metabolic dysfunction. Additionally, we aim to identify gaps in the current research and highlight the priorities for future high-quality randomized controlled trials.

## 2. Materials and Methods

### 2.1. Study Protocol and Registration

To conduct this systematic review and meta-analysis [21], we used the Preferred Reporting Items for Systematic Reviews and Meta-Analysis (PRISMA) guidelines. We followed all the steps mentioned in Cochrane’s Handbook of Systematic Reviews of Interventions [22]. The study protocol was registered on the Open Science Framework (OSF) under the following DOI: https://doi.org/10.17605/OSF.IO/VR428.

### 2.2. Search Strategy and Data Collection

We searched four electronic databases: Scopus, PubMed, Web of Science (WoS), and Medline via WOS. We searched for all studies published until February 2025. We used the terms indicated in Supplementary File S1 and Supplementary Table S1. We included all types of primary studies, such as cross-sectional studies, randomized clinical trials, and cohort studies, except case reports and case series, which assessed the use of lycopene in relation to metabolic diseases such as metabolic syndrome, obesity, DM, insulin resistance, or MAFLD. We included only English studies published between 2001 and 2025.

### 2.3. Eligibility Criteria

Inclusion Criteria:

Population: Adults (≥18 years) with or without metabolic dysfunction (e.g., MAFLD, obesity, diabetes, and insulin resistance).
Intervention/Exposure: Serum lycopene levels or lycopene supplementation.
Comparison: Healthy individuals or placebo/control groups.
Outcomes: Metabolic markers (IGF-1, IGFBPs, glucose, insulin, and lipids), MAFLD risk, obesity, and diabetes status.
Study Design: RCTs, cohort studies, case–control studies, and cross-sectional studies.
Language: English.
Publication Date: 2001 to 2025.

Exclusion Criteria:

Case reports, case series, reviews, and editorials were excluded, as were animal or in vitro studies.

We removed duplicates using EndNote Software Version (X-9). We assessed all the retrieved studies according to our eligibility criteria in two steps. Firstly, we conducted title and abstract screening; then, we screened the full texts of the retrieved studies. Studies that met our eligibility criteria were included. Each screening step was independently conducted by two distinct reviewers. Discrepancies were resolved through consensus or adjudication by a third reviewer.

### 2.4. Effect Estimates and Data Extraction for Meta-Analysis

Two authors extracted data using two Excel sheets, a summary and baseline sheet and an outcome sheet, and a third author resolved any conflicts.

For dichotomous outcomes (e.g., risk of MAFLD, diabetes history, and mortality), we extracted odds ratios (ORs) and hazard ratios (HRs), preferentially selecting the most fully adjusted estimates reported in the original studies to minimize confounding bias. When multiple adjusted models were presented, we consistently extracted estimates from the model with the greatest number of covariates.

For continuous outcomes (e.g., IGF-1, IGFBPs, and serum lycopene levels), we extracted mean differences (MDs) and corresponding standard deviations (SDs) or standard errors (SEs). When available, adjusted mean values were prioritized; otherwise, unadjusted means were used for the analysis. In cases where studies reported medians and interquartile ranges, we applied validated methods to estimate the means and SDs when appropriate.

Pooled estimates were calculated using either fixed- or random-effects models, depending on the degree of statistical heterogeneity, as assessed via the I^2^ and τ^2^ statistics. Sensitivity analyses and publication bias assessments were conducted for outcomes with at least three contributing studies.

### 2.5. Risk-of-Bias Assessment

We evaluated the methodological rigor of the included studies using two distinct tools. RCTs were assessed using the Cochrane RoB 2 framework (Sterne et al., 2019) [23]. This approach is used to examine potential bias across five key areas: randomization procedures (D1), protocol adherence (D2), data completeness (D3), outcome measurements (D4), and selective reporting (D5). Our team assigned “Low risk”, “Some concerns”, or “High risk” ratings to each domain and then made comprehensive judgments for each study, as recommended by Higgins et al. (2022) [24].

Non-randomized investigations required a different approach; therefore, we selected the ROBINS-I instrument (Sterne et al., 2016) [25] for these assessments. This nuanced tool scrutinizes seven potential bias sources: confounding variables (D1), participant selection methods (D2), intervention classification (D3), protocol deviations (D4), missing information (D5), outcome measurement techniques (D6), and selective result-reporting (D7). For these studies, we applied “Low”, “Moderate”, “Serious”, or “Critical” risk designations across domains, with the most concerning rating determining the overall assessment, in accordance with established guidance (Page et al., 2021) [26].

Two team members independently conducted these evaluations and compared their findings. Whenever disagreements emerged, we held thorough discussions and occasionally consulted a third colleague to resolve stalemates. While no studies faced exclusion based solely on these quality metrics, our risk assessments substantially shaped our interpretation of the findings and informed our sensitivity analysis strategy in accordance with current best practices (Boutron et al., 2019) [27].

### 2.6. Data Analysis

Meta-analyses were performed using R (v. 4.4.0.), employing the ‘meta’ and ‘metafor’ packages. For each analysis, both fixed-effects and random-effects models were applied, with the appropriate model selected based on heterogeneity assessment. Heterogeneity was quantified using the I^2^ statistic, with values >50% indicating substantial heterogeneity, in which case random-effects models were preferred. The magnitude of I^2^ was interpreted according to the Cochrane guidelines [28]. The Hartung–Knapp adjustment was applied when calculating CIs and *p*-values for pooled estimates using metagen or metacont. The DerSimonian and Laird method was used for estimating between-study variance (τ^2^) in random-effects models [29,30,31,32].

For continuous outcomes (serum lycopene levels, IGF-I, IGF-II, IGFBP-1, IGFBP-2, and IGFBP-3 levels), mean differences with 95% confidence intervals were calculated. For binary outcomes (MAFLD risk, mortality risk, high HbA1c, and history of diabetes), odds ratios (ORs) or hazard ratios (HRs) with 95% confidence intervals were computed using inverse variance weighting.

### 2.7. Planned Subgroup Analysis

Subgroup analyses were conducted to examine the differences between populations (e.g., diabetes vs. control groups, no metabolic syndrome vs. metabolic syndrome, etc.). Sensitivity analyses were performed using leave-one-out methods to assess the influence of individual studies on the pooled estimates. Publication bias was evaluated using funnel plots, although their interpretation was limited to analyses with few studies.

For studies reporting dietary lycopene intake, a meta-analysis of raw means (MRAW) was performed. For the comparison between the diabetes and control groups, separate single-arm meta-analyses were first performed, followed by subgroup difference analysis to determine statistical significance.

Statistical significance was set at *p* < 0.05 for all analyses. Forest plots were generated to visualize the individual study effects and pooled estimates, with diamonds representing the overall effect sizes and their 95% confidence intervals.

## 3. Results

### 3.1. Study Selection

**Figure 1:**
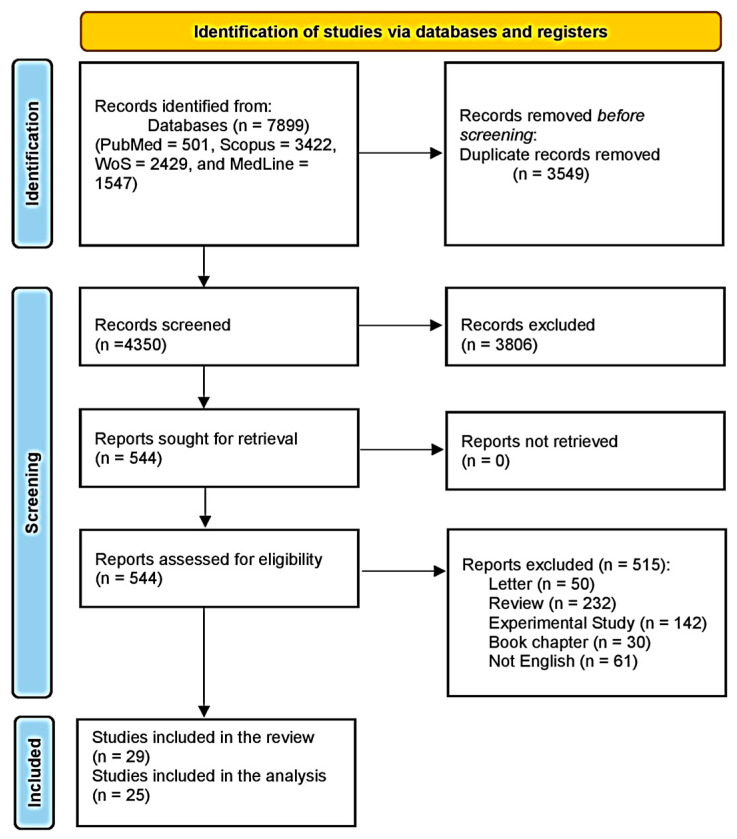
Study-selection flow diagram (Preferred Reporting Items for Systematic Reviews and Meta-Analyses).

A literature search conducted using PubMed, Scopus, WoS, and Medline revealed 7899 articles, 3549 of which were duplicates. Title and abstract screening were performed for 4350 articles, and 3806 were excluded. We then screened the full texts of 544 studies. Finally, 29 studies were included in this systematic review, 25 of which were included in the meta-analysis. A flow diagram of this process is shown in Figure 1.

### 3.2. Characteristics of the Included Studies

Twenty-nine studies from diverse geographical regions, including the United States, China, Japan, Australia, the United Kingdom, Israel, Malaysia, the Netherlands, Finland, and Canada, were included in this review [33,34,35,36,37,38,39,40,41,42,43,44,45,46,47,48,49,50,51,52,53,54,55,56,57,58,59,60]. The included studies were published between 2002 and 2024 and encompassed various study designs, including cross-sectional studies, randomized clinical trials, prospective cohort studies, case–control studies, and secondary analyses of clinical trials.

Most of the studies (n = 17) were cross-sectional in design and primarily investigated the association between metabolic diseases, particularly MAFLD, obesity, metabolic syndrome, hyperglycemia, and various outcomes. Four prospective cohort studies and six randomized clinical trials also contributed to the evidence base, focusing largely on metabolic dysregulation and IGF decline in older adults. Sample sizes varied widely across studies, ranging from as few as 8 participants in a clinical trial [47] to more than 30 million individuals in a large US cross-sectional analysis [29]. The largest cross-sectional studies included 91,752,219 [40], 16,191 [53], and 25,868 participants [36].

Randomized clinical trials focusing on IGF decline enrolled smaller cohorts, typically fewer than 100 participants [39,50,52]. The prospective cohort studies provided long-term data on hyperglycemia and its related outcomes [57,59]. The included studies collectively examined a wide range of populations across different age groups, healthcare systems, and ethnic backgrounds. Table 1 summarizes the characteristics of the included studies, including their settings, study designs, the types of metabolic disease investigated, and sample sizes.

### 3.3. Baseline Characteristics of the Included Patients

The demographic and clinical characteristics of the patients in the included studies vary widely. The mean age of the participants ranged from 12.7 [41] to 65 years [39]. Cases were generally older than controls in studies such as those conducted by Chai et al. (2024) (51.4 ± 0.7 vs. 46.7 ± 0.7 years) and Christensen et al. (2019) [33,40]. The proportion of males was consistently higher among the cases than among the controls. For instance, Chai et al. (2024) [33] reported 61.8% male cases and 45.7% male controls, and Christensen et al. (2019) [40] noted 54.7% of cases were males, while this figure was 42.4% among controls [32,39]. Educational attainment varied between the cases and controls. In Chai et al.’s study (2024), the proportion of participants with an education level above high school was lower among cases (25.6%) than among controls (33.2%) [33]. Similarly, Christensen et al. (2019) found that cases were more likely to have lower educational levels than controls [40].

Data on physical activity were reported in some of the studies. Yu et al. (2024) reported that cases had lower levels of physical activity, with 22% classified as having low activity compared to 14.3% among controls [35]. Similarly, Zhang et al. (2023) reported a higher proportion of cases with low physical activity (16.65%) in comparison to that for controls (9.19%) [37].

Across studies, there was generally a higher proportion of current or former smokers among cases in comparison to that for controls. For example, Chai et al. (2024) reported that 48.9% of cases were either current or former smokers, while this value was 41.2% among controls [33]. Yu et al. (2023) reported that the percentage of current smokers was slightly higher among controls, although the overall patterns showed a greater smoking burden among cases [36]. The differences in alcohol consumption were inconsistent. Yu et al. (2024) found that a greater proportion of controls were categorized as drinkers or heavy drinkers compared to cases [35]. Similarly, Suzuki et al. (2011) detailed patterns by sex, noting greater alcohol use among male participants [45].

Obesity and total cholesterol levels have been inconsistently reported. Cases were generally more obese than controls in most of the studies reporting Body Mass Index (BMI) values. For instance, in the study by Zhang et al. (2023), 62.33% of cases were classified as obese, while 17.75% of the controls were classified as such [37]. Chai et al. (2024) also found higher BMI and triglyceride levels among cases ((34.9 ± 0.5) and (150 ± 9.3)) in comparison to those for controls ((27.6 ± 0.3) and (99.4 ± 2.2), respectively) [33]. Similarly, Suzuki et al. (2011) reported slightly higher cholesterol levels in females than in males [45]. Triglyceride levels were elevated in several studies. Lin et al. (2024) found mean triglyceride levels of 2.26 ± 0.06 among cases; these values can be compared to the 1.6 ± 0.6 found among controls in Leh et al. (2021) [34,38]. Several studies did not report baseline characteristics in detail, which limited cross-study comparisons. Supplementary Table S2 presents the characteristics of the included patients.

### 3.4. Risk of Bias

**Figure 2:**
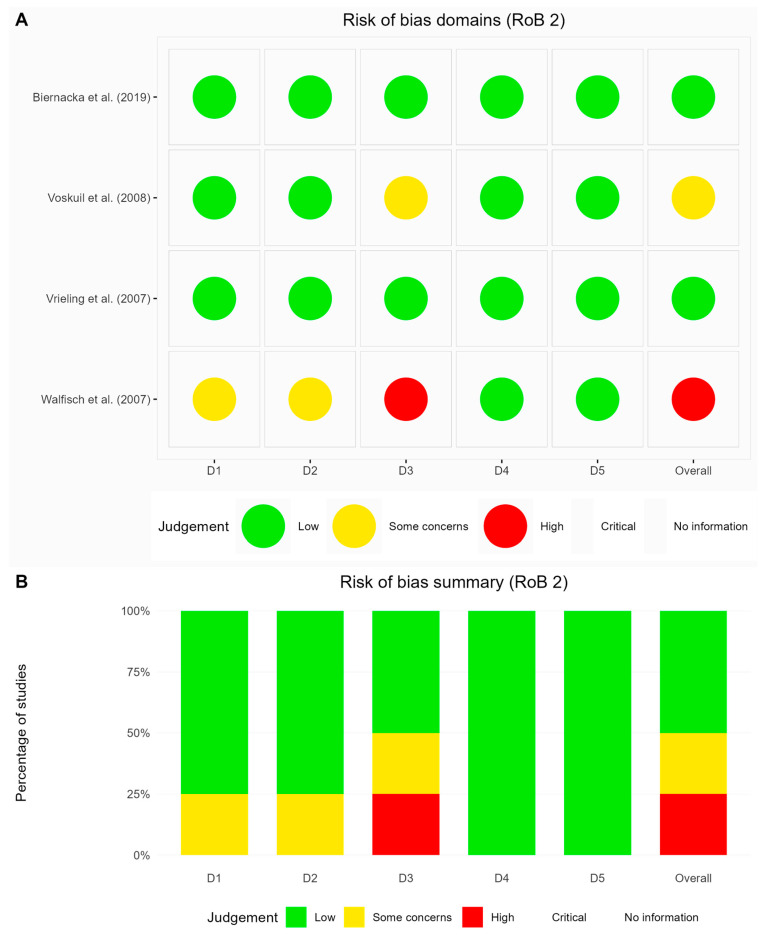
Assessment of the included RCTs via the Cochrane RoB 2 framework. (**A**) Risk-of-bias domains [39,50,51,52] and (**B**) risk-of-bias summary.

Our evaluation of methodological quality revealed considerable limitations across the 29 included studies. Among randomized controlled trial (RCTs), Biernacka et al. [39] and Vrieling et al. [51] demonstrated methodological excellence with consistently low risk ratings, whereas Voskuil et al. [50] showed minor deficiencies in outcome data completeness, and Walfisch et al. [52] exhibited significant methodological weaknesses across several domains (Figure 2).

**Figure 3:**
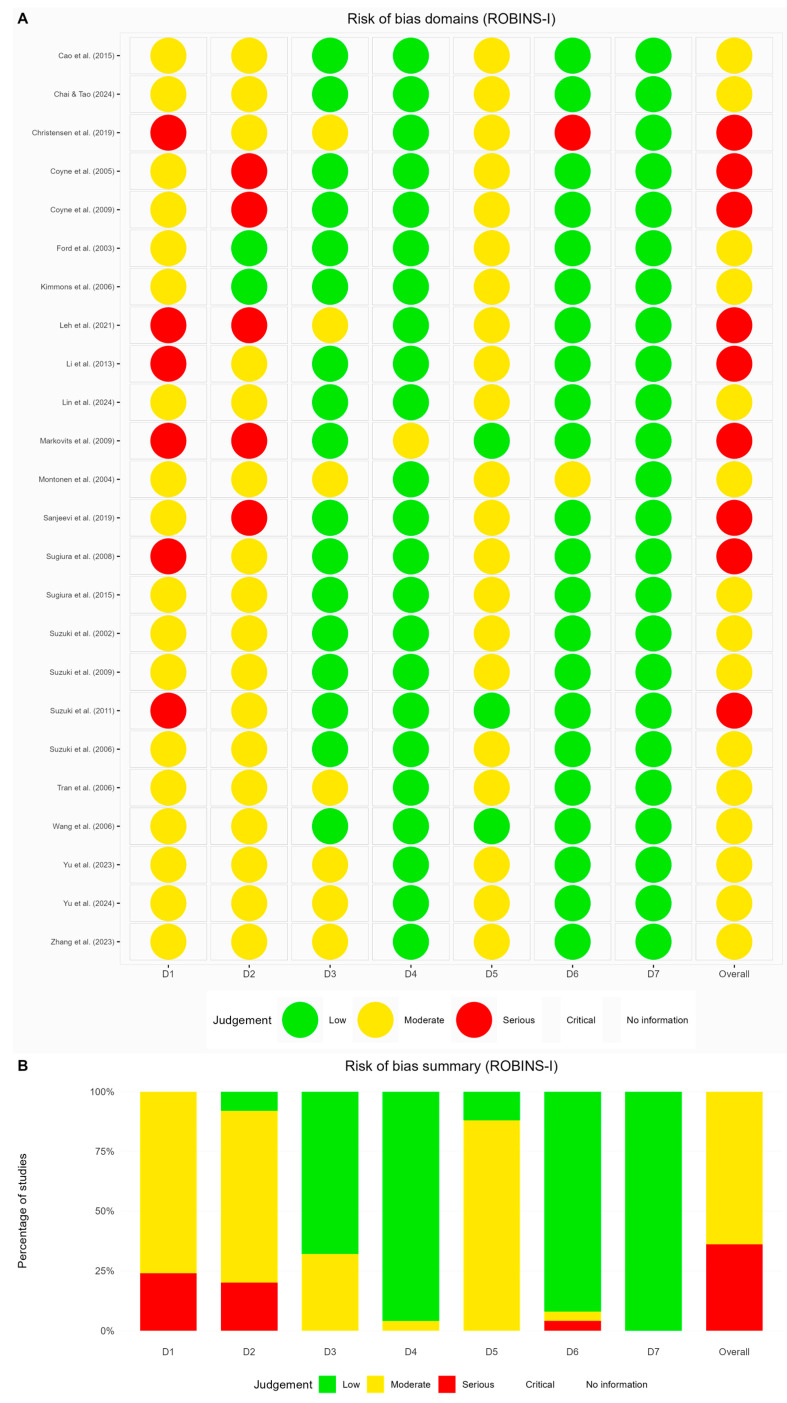
Assessment of non-randomized investigations via the ROBINS-I instrument: (**A**) risk-of-bias domains [33,34,35,36,37,38,40,41,42,43,44,45,46,47,48,49,53,54,55,56,57,58,59,60] and (**B**) risk-of-bias summary.

Non-randomized studies presented even greater concerns; of the 25 studies assessed via ROBINS-I, 16 demonstrated moderate risk of bias, while 9 reached serious risk thresholds (Figure 3). Notably, confounding variables and participant selection emerged as particularly problematic aspects across the literature. The comprehensive assessment indicated that merely 7% of studies achieved low-risk-of-bias status, with 90% exhibiting moderate to serious methodological shortcomings—a finding that reveals the need for cautious interpretation of the existing evidence in this field.

### 3.5. Primary and Secondary Outcomes

#### 3.5.1. Serum Lycopene Levels and MAFLD Risk

**Figure 4:**
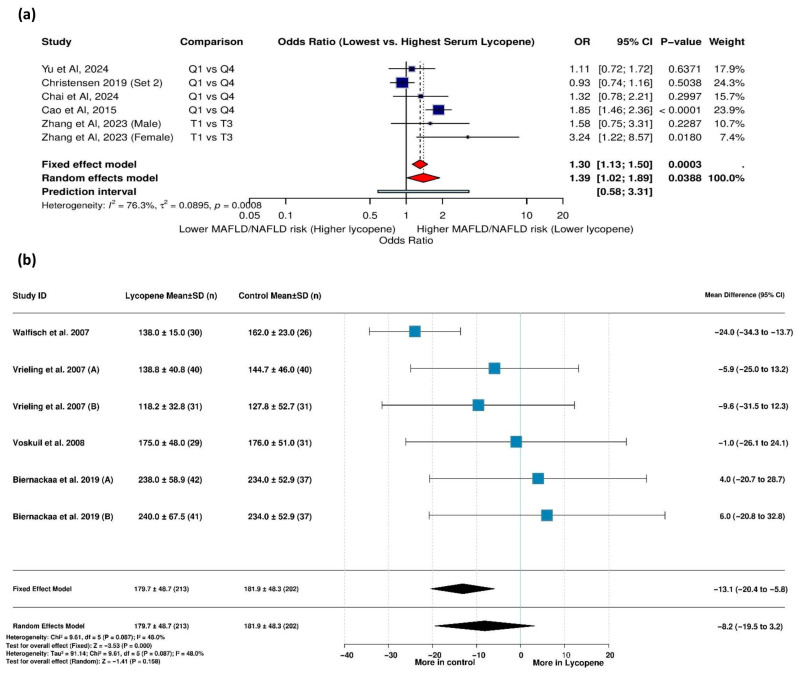
Forest plot showing the effects of lycopene on (**a**) MAFLD risk and (**b**) IGF-I levels (ng/mL). Vrieling et al. (2007) (A) [51] assessed the effect of lycopene versus a control on IGF-I levels in men, while Vrieling et al. (2007) (B) [51] assessed the effect of lycopene versus a control on IGF-I levels in women. Biernackaa et al. (2019) (A) [39] assessed the effect of a lycopene-rich diet vs. a placebo on IGF-I levels. Biernackaa et al. (2019) (B) [39] assessed the effect of lycopene supplementation vs. a placebo on IGF-I levels.

The meta-analysis examining the relationship between serum lycopene levels and MAFLD risk included six studies with substantial heterogeneity (I^2^ = 76.3%, τ^2^ = 0.0895, *p* = 0.0008), necessitating the use of a random-effects model. The results revealed that individuals with the lowest serum lycopene levels had a significantly higher risk of MAFLD compared to those with the highest levels (OR = 1.39 [95% CI: 1.02, 1.89], *p* = 0.0388) (Figure 4a).

Sensitivity analysis showed that removing the study by Christensen (2019) reduced heterogeneity considerably (I^2^ decreased from 76.3% to 39.4%) while strengthening the association (OR = 1.57 [1.20; 2.07], *p* = 0.0012), suggesting this study had a moderating effect on the overall findings (Figure S1).

Summary: Lower serum lycopene levels are consistently associated with increased MAFLD risk. Sensitivity analyses confirmed this relationship remains significant after addressing heterogeneity.

#### 3.5.2. Lycopene Supplementation and IGF-I Levels

Six studies with 415 participants were included in the analysis of IGF-I levels between the lycopene supplementation and control groups. With moderate heterogeneity present (I^2^ = 48.0%, *p* = 0.087), a fixed-effects model showed there were significantly lower IGF-I levels in the lycopene group than in the control group (MD = −13.1 ng/mL [95% CI: −20.4, −5.8], *p* < 0.001) (Figure 4b). The results of a leave-one-out sensitivity analysis identified Walfisch et al. (2007) [39] as being particularly influential; removing this study eliminated heterogeneity (I^2^ = 0.0%) and rendered the effect insignificant, suggesting that this study largely drove the observed effect (Figure S2).

Summary: Lycopene supplementation may reduce IGF-I levels; however, sensitivity analysis revealed the veracity of this effect depends heavily on a single study, indicating the need for further confirmatory research.

#### 3.5.3. IGF-I Levels Across Lycopene Quantiles

**Figure 5:**
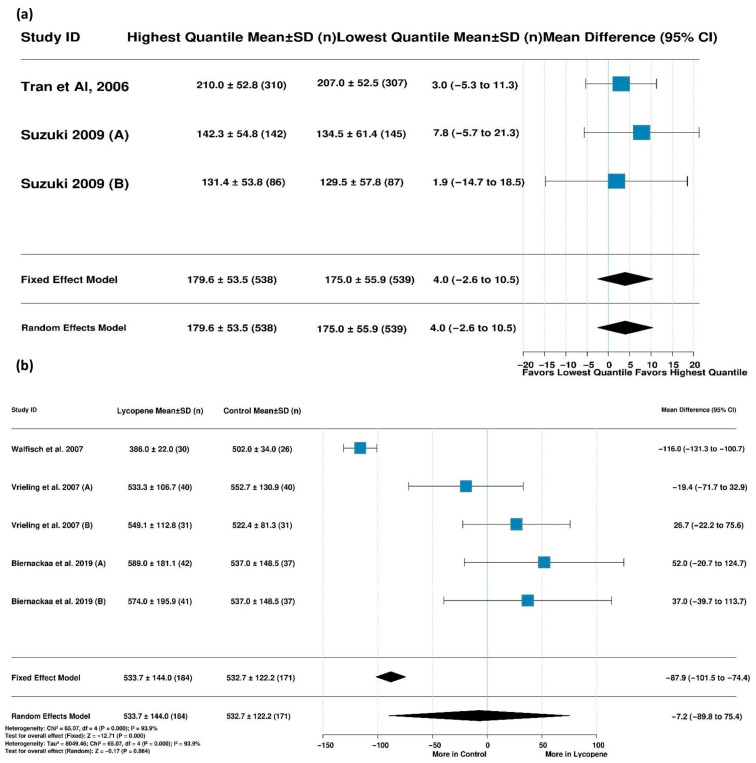
Forest plots of lycopene’s effects on IGF-1 and IGF-II levels. (**a**) Effect of lycopene on IGF-I levels regarding highest vs. lowest lycopene quantiles [48,61] and (**b**) mean difference in IGF-II levels: lycopene vs. control [39,51,52]. Suzuki et al. (2009) (A) [48] assessed the effect of lycopene versus a control in men, while Suzuki et al. (2009) (B) [48] assessed the effect of lycopene versus a control in women. Vrieling et al. (2007) (A) [51] assessed the effect of lycopene versus a control in men, while Vrieling et al. (2007 (B) [51] assessed the effect of lycopene versus a control in women. Biernackaa et al. (2019) (A) [39] assessed the effect of a lycopene-rich diet vs. a placebo. Biernackaa et al. (2019) (B) [39] assessed the effect of lycopene supplementation vs. a placebo.

The analysis comparing IGF-I levels between individuals with the highest and lowest lycopene quantiles included three studies with 1077 participants. With no heterogeneity detected (I^2^ = 0.0%, *p* = 0.809), the fixed-effects model showed no significant differences in IGF-I levels between the highest and lowest lycopene quantiles (Figure 5a). Sensitivity analyses in which individual studies were removed did not substantially alter the results, confirming the robustness of this finding across all the studies included (Figure S3).

Summary: Based on current evidence, circulating IGF-I levels do not differ significantly between individuals with high versus low lycopene concentrations.

#### 3.5.4. Lycopene Supplementation and IGF-II Levels

Five studies with 355 participants contributed to the analysis of IGF-II levels. High heterogeneity was observed (I^2^ = 93.9%, *p* < 0.0001), warranting the use of a random-effects model. The analysis found no significant differences in IGF-II levels between the lycopene supplementation and control groups (Figure 5b).

The leave-one-out analysis revealed that the study by Walfisch et al. [52] (2007) substantially influenced heterogeneity; its removal dramatically reduced the I^2^ from 93.9% to 5.0% and shifted the effect estimate to MD = 17.9 [−12.6 to 48.5], though the corresponding difference still did not reach statistical significance (Figure S4).

Summary: There is no conclusive evidence that lycopene supplementation affects IGF-II levels; the high heterogeneity is explained by a single influential study.

#### 3.5.5. Lycopene Supplementation and IGFBP-1 Levels

**Figure 6:**
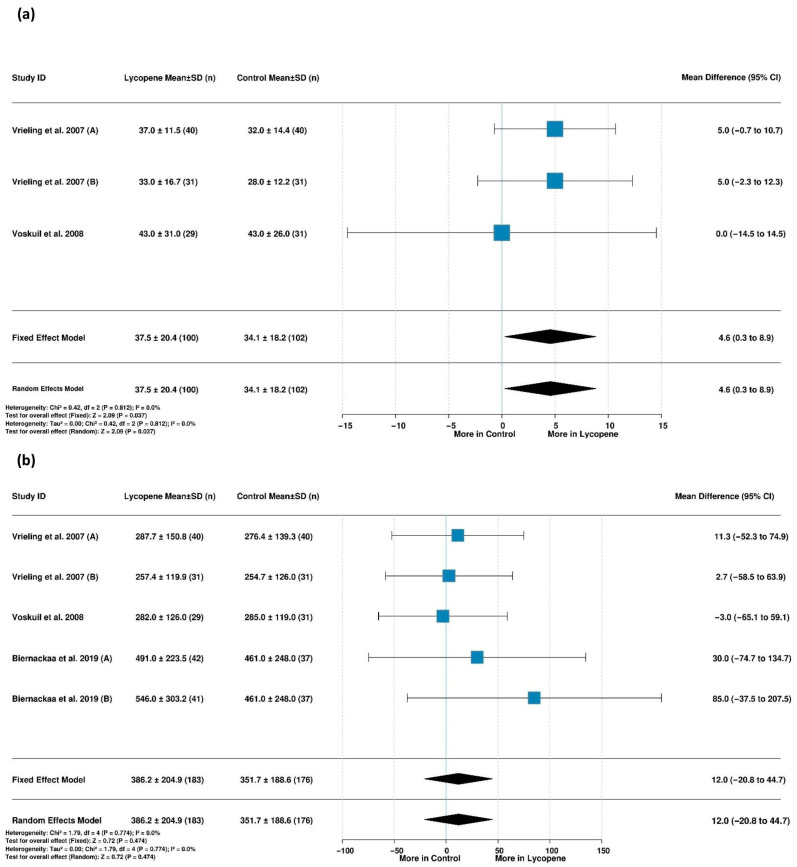
Forest plots showing lycopene’s effects on (**a**) IGFBP-1 levels [50,51] and (**b**) IGFBP-2 levels [39,50,51]. Vrieling et al. (2007) (A) [51] assessed the effect of lycopene versus a control in men, while Vrieling et al. (2007) (B) [51] assessed the effect of lycopene versus a control in women. Biernackaa et al. (2019) (A) [39] assessed the effect of a lycopene-rich diet vs. a placebo. Biernackaa et al. (2019) (B) [39] assessed the effect of lycopene supplementation vs. a placebo.

Three studies involving a total of 202 participants were analyzed for differences in insulin-growth-factor-binding protein-1 (IGFBP-1) levels. No heterogeneity was detected (I^2^ = 0.0%, *p* = 0.812); the fixed-effects model demonstrated significantly higher IGFBP-1 levels in the lycopene group than in the control group (MD = 4.6 [95% CI: 0.3, 8.9], *p* = 0.037) (Figure 6a). Sensitivity analysis showed that removing Voskuil et al.’s study (2008) [50] strengthened the association (MD = 5.0 [0.5 to 9.5], *p* < 0.05), while the effect remained consistent and significant regardless of which study was excluded, indicating the stability of the findings (Figure S5).

Summary: Lycopene supplementation appears to increase IGFBP-1 levels, with consistent findings across studies.

#### 3.5.6. Lycopene Supplementation and IGFBP-2 Levels

The analysis of IGFBP-2 levels included five studies with 359 participants. In the absence of heterogeneity (I^2^ = 0.0%, *p* = 0.774), the fixed-effects model showed no significant difference in IGFBP-2 levels between the lycopene and controls. The leave-one-out sensitivity analyses confirmed the robustness of this finding, as the removal of individual studies did not meaningfully alter the effect estimate or its statistical significance, suggesting there were consistent results across the included studies (Figure S6).

Summary: Current data do not support the notion that lycopene supplementation has a noticeable effect on IGFBP-2 levels.

#### 3.5.7. Lycopene Supplementation and IGFBP-3

**Figure 7:**
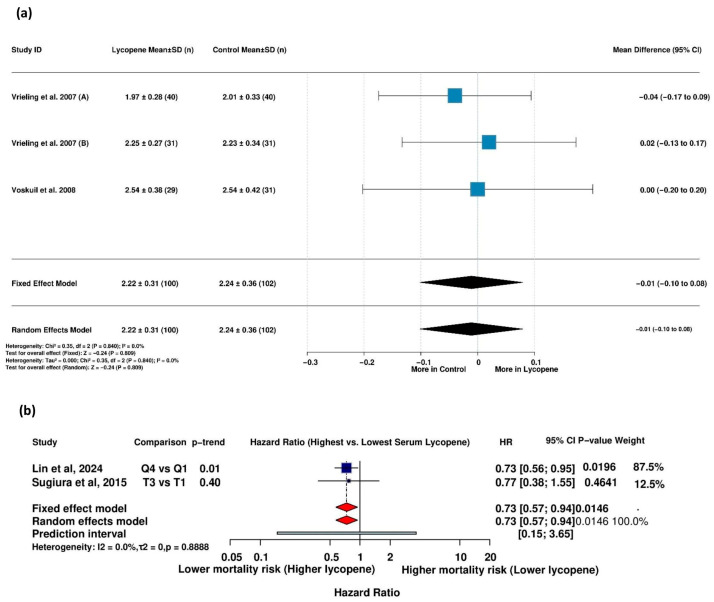
Association between lycopene and IGFBP-3 levels and mortality in relation to metabolic disease. (**a**) Impact of lycopene supplementation vs. a control on IGFBP-3 levels (mean difference) [50,51]. (**b**) Association between serum lycopene levels and mortality among patients with metabolic diseases (hazard ratios) [34,49]. Vrieling et al. (2007) (A) [51] assessed the effect of lycopene versus a control in men, while Vrieling et al. (2007) (B) [51] assessed the effect of lycopene versus a control in women.

Three studies with 202 participants were analyzed regarding differences in IGFBP-3 levels between lycopene and control groups. No heterogeneity was observed (I^2^ = 0.0%, *p* = 0.840), and the fixed-effects model found no significant difference in IGFBP-3 levels between the groups (Figure 7a). The consistency of this finding was confirmed through sensitivity analyses, wherein excluding individual studies did not substantially change the effect estimate or its statistical significance, indicating reliable results across the dataset (Figure S7).

Summary: Lycopene supplementation does not significantly alter IGFBP-3 levels. The absence of heterogeneity and consistent results in sensitivity analyses suggests a robust and reliable null effect across the included studies.

#### 3.5.8. Association Between Serum Lycopene and Mortality in Patients with Metabolic Diseases

The meta-analysis examining the relationship between serum lycopene levels and mortality risk among patients with metabolic diseases included two studies with no observed heterogeneity (I^2^ = 0.0%, τ^2^ = 0, *p* = 0.8888). Via a fixed-effects model, the analysis found that higher serum lycopene levels were significantly associated with a lower mortality risk (HR = 0.73 [95% CI: 0.57, 0.94], *p* = 0.0146) (Figure 7b). The study by Lin et al. (2024) [34] contributed substantially to this finding, accounting for 87.5% of the overall weight, which suggests the result is heavily influenced by this single study despite the consistent direction of the effect across both the included studies.

Summary: Higher serum lycopene levels are associated with a reduced mortality risk among metabolic disease patients. However, this conclusion is primarily driven by one large study, warranting cautious interpretation despite consistent effect directions.

#### 3.5.9. Association Between Serum Lycopene and High HbA1c Levels

Two studies investigating the association between serum lycopene and high HbA1c levels were analyzed. Substantial heterogeneity was detected (I^2^ = 72.2%, τ^2^ = 0.1692, *p* = 0.0577), necessitating a random-effects model. The substantial heterogeneity may reflect the divergent findings between the two studies, with Suzuki et al. (2007) [54] reporting a protective effect (OR = 0.58), while Wang et al. (2006) [56] found the opposite (OR = 1.15), highlighting the uncertainty in this relationship.

Summary: Current evidence does not demonstrate a clear association between serum lycopene and elevated HbA1c levels. Substantial heterogeneity and conflicting study results highlight uncertainty and the need for further research.

#### 3.5.10. Association Between Serum Lycopene and History of Diabetes Mellitus

**Figure 8:**
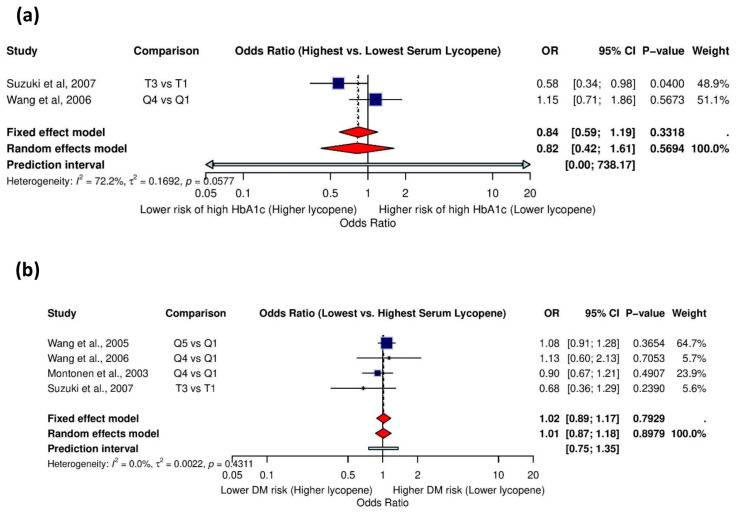
Association between serum lycopene levels and glycemic outcomes: (**a**) odds ratios for the association between serum lycopene and high HbA1c levels [54,56], and (**b**) odds ratios for the association between serum lycopene and history of *diabetes mellitus* [54,56,57,59].

Four studies were included in the analysis of the relationship between serum lycopene levels and a history of *diabetes mellitus*. With no detected heterogeneity (I^2^ = 0.0%, τ^2^ = 0.0022, *p* = 0.4311), the fixed-effects model found no significant association between serum lycopene levels and diabetes history (Figure 8b). The sensitivity analyses confirmed the stability of this finding, as removing individual studies did not substantially alter the effect estimate or statistical significance, suggesting consistent null results across the included studies (Figure S8).

Summary: There is no significant association between serum lycopene levels and a history of diabetes mellitus. The consistent findings across studies and lack of heterogeneity support the robustness of this null result.

#### 3.5.11. Mean Difference in Serum Lycopene Levels: Diabetic vs. Control

**Figure 9:**
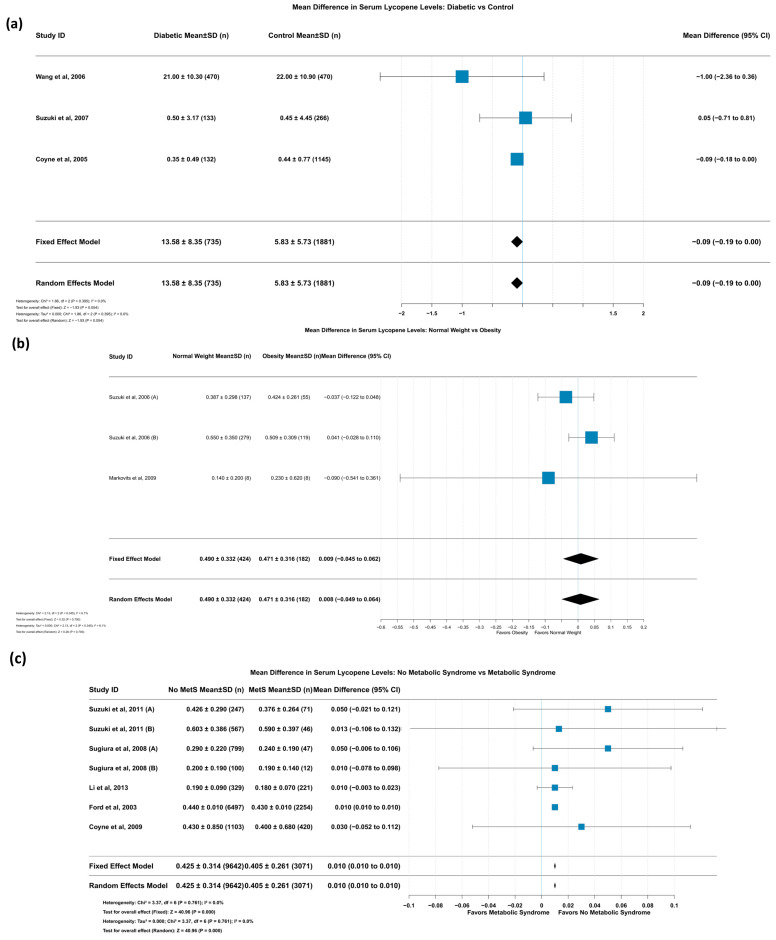
Differences in serum lycopene levels according to metabolic status. (**a**) Mean difference in serum lycopene levels between diabetic and non-diabetic individuals [48,56,58]. (**b**) Mean difference in serum lycopene levels between normal-weight and obese individuals [47,54] and (**c**) mean difference in serum lycopene levels between individuals without metabolic syndrome versus those with the disease [44,45,46,49,60]. Suzuki et al. (2006) (A) [54] assessed the effect of lycopene in men, while Suzuki et al. (2009) (B) [48] assessed the effect of lycopene in women. Suzuki et al. (2011) (A) [45] assessed the effect of lycopene in men, while Suzuki et al. (2011) (B) [45] assessed the effect of lycopene in women. Sugiura et al. (2008) (A) [49] assessed the effect of lycopene in non-smokers, while Sugiura et al. (2008) (B) [49] assessed the effect of lycopene in smokers.

Three studies with 2616 participants were analyzed to compare serum lycopene levels between patients with diabetes and controls. No heterogeneity was observed (I^2^ = 0.0%, *p* = 0.395); the fixed-effects model found a borderline-significant trend toward lower serum lycopene levels in patients in comparison to controls (MD = −0.09 [95% CI: −0.19, 0.00], *p* = 0.054) (Figure 9a).

The leave-one-out sensitivity analysis showed that removing Coyne et al.’s study (2005) [45] increased heterogeneity (I^2^ = 43.0%), suggesting some variability in effect size across studies, though the direction of the effect remained consistent (Figure S9).

Summary: There is a borderline trend suggesting there are lower serum lycopene levels in diabetic individuals compared to those in controls, though the evidence is not conclusive. Sensitivity analyses indicate some variability but reveal a consistent direction across studies.

#### 3.5.12. Mean Difference in Serum Lycopene Levels: Normal Weight vs. Obesity

Three studies involving a total of 606 participants compared serum lycopene levels between normal-weight and obese individuals. With minimal heterogeneity detected (I^2^ = 6.1%, *p* = 0.345), the fixed-effects model found no significant difference in serum lycopene levels between the weight groups (Figure 9b).

Sensitivity analysis revealed that removing the study by Markovits et al. (2009) [47] increased heterogeneity to I^2^ = 48.6%, indicating that this study had a stabilizing effect on the overall results, although the effect estimate remained non-significant regardless of which study was excluded (Figure S10).

Summary: Serum lycopene levels do not differ significantly between normal-weight and obese individuals. The corresponding findings are stable across studies, with minimal heterogeneity.

#### 3.5.13. Mean Difference in Serum Lycopene Levels: No Metabolic Syndrome vs. Metabolic Syndrome

Seven studies with a substantial sample size of 12,713 participants contributed to the analysis comparing serum lycopene levels between individuals with and without metabolic syndrome. With no heterogeneity observed (I^2^ = 0.0%, *p* = 0.761), the fixed-effects model demonstrated significantly higher serum lycopene levels in individuals without metabolic syndrome compared to those with the condition (MD = 0.010 [95% CI: 0.010, 0.010], *p* < 0.001) (Figure 9c).

This finding remained robust in sensitivity analyses, with the removal of the study by Ford et al. (2003) [60] slightly increasing the effect size (MD = 0.014 [0.001 to 0.026]) without introducing heterogeneity, affirming the consistency of this relationship across studies.

Summary: Higher serum lycopene levels are significantly associated with the absence of metabolic syndrome. The relationship is consistent and robust across multiple large studies, showing no heterogeneity.

### 3.6. Publication Bias

Publication bias was assessed using funnel plots for outcomes from three or more studies. Visual inspection of the funnel plots revealed asymmetrical distributions for several lycopene-related outcomes.

For the MAFLD risk analysis, the funnel plots showed moderate asymmetry, with smaller studies (particularly Zhang et al.’s (2023) [37]) reporting stronger protective effects. In the IGF-I analysis, the funnel plot demonstrated notable asymmetry, with Walfisch et al.’s study (2007) [52] appearing as an outlier with stronger negative effects. Similarly, the IGF-II levels comparison displayed marked asymmetry, with the study by Walfisch et al. (2007) [52] again appearing as a distinct outlier, corresponding with extremely high heterogeneity (I^2^ = 93.9%).

For the IGFBP-1, IGFBP-2, and IGFBP-3 analyses, the funnel plots showed relatively symmetrical distributions, suggesting minimal publication bias for these outcomes. The diabetes-related analyses displayed variable patterns, with the diabetes history funnel plot appearing reasonably balanced, while the plots for normal weight vs. obesity and metabolic syndrome comparisons showed more clustered distributions. However, interpretation of these funnel plots should be cautiously approached due to the limited number of studies for most outcomes and the high heterogeneity observed in several analyses (particularly IGF-II with I^2^ = 93.9% and HbA1c with I^2^ = 72.2%) (Figures S11–S13).

## 4. Discussion

This systematic review and meta-analysis comprehensively evaluated the relationship between serum lycopene levels and various metabolic outcomes by synthesizing data from 29 studies conducted across diverse global regions. The included studies, published between 2002 and 2024, had multiple different designs, with sample sizes ranging from very small trials to analyses involving millions of participants. Despite the heterogeneity of the populations, settings, and methodologies, several important patterns emerged.

### 4.1. Association Between Serum Lycopene and MAFLD Risk

First, our findings showed that high serum lycopene levels were significantly associated with a decreased risk of MAFLD. This highlights the potential role of lycopene in the prevention of metabolic liver disease. These results are consistent with the findings of Chai et al. (2024), who reported that higher levels of serum carotenoids, including lycopene, were associated with reduced odds of metabolic dysfunction-associated steatotic liver disease (MASLD) [33]. Additionally, Christensen et al. (2019) reported that serum carotenoids, including lycopene and lutein combined with zeaxanthin, were inversely correlated with the likelihood of nonalcoholic fatty liver disease (NAFLD) based on the NHANES 2003–2014 data, although liver steatosis status was not assessed using objective transient elastography measures in their study [40].

Serum and plasma carotenoid concentrations are influenced not only by an individual’s dietary consumption of these compounds but also by several physiological variables. Studies have indicated that carotenoid levels are lower in obese individuals relative to those of normal-weight individuals, which may be attributable to elevated subcutaneous fat and heightened oxidative stress linked to obesity [62,63]. Chai et al. (2024) revealed that the correlations between dietary and serum levels of micronutrients were more pronounced for carotenoids (α-carotene, r = 0.34; β-carotene, r = 0.31; r = 0.29; lycopene, r = 0.25) than for other micronutrients such as α-tocopherol (r = 0.10) and retinol (r = 0.05), indicating that the serum levels of carotenoids partially reflect their dietary intake [33]. The associations between food consumption and blood concentrations of micronutrients were consistent among individuals with and without MASLD. This may partially elucidate the observed beneficial correlations between steatotic liver diseases (SLDs) and both dietary and serum β-carotene, as well as the protective link with dietary vitamin E (α-tocopherol), although no such association was discovered for serum α-tocopherol, which exhibited a positive correlation in men [64].

Sex is believed to significantly influence liver disease and other metabolic illnesses. The prevalence of MASLD was markedly greater in men (34.1%) than in women (21.2%). Consequently, gender-specific preventive and treatment measures are essential to reduce the prevalence of MASLD. Chai et al. (2024) [33] performed sex-stratified analyses and revealed that men and women exhibited comparable results regarding the relationships between serum micronutrients and MASLD, except α-tocopherol, which demonstrated a significant link only in men. Additionally, the cited authors revealed that age significantly contributed to MASLD, as people with MASLD were older than their counterparts without the disease, regardless of their sex. Consequently, they controlled for participants’ age (as a continuous variable/covariate) in their analytical models to eliminate the potential impact of age on the associations between micronutrients and MASLD [40].

The recently established MASLD illustrates the significant epidemiological and pathological connections among NAFLD, metabolic dysfunction, and insulin resistance. The role of alcohol use in SLDs is now recognized as having overlapping molecular pathways that contribute to both NAFLD and alcohol-related liver disease (ALD). Chai et al. (2024) [33] accounted for participants’ daily alcohol use and drinking patterns in their analyses and conducted a stratified analysis according to the Delphi criteria [65] for low (F: <20 g/day; M: <30 g/day) and moderate alcohol intake (F: 20–50 g/day; M: 30–60 g/day). The associations between serum micronutrients and MASLD exhibited similar trends for most micronutrients (e.g., γ-tocopherol, 25(OH)D, α-carotene, β-carotene, α-cryptoxanthin, β-cryptoxanthin, and lutein and zeaxanthin combined) across both subgroups (low- and moderate-level alcohol consumers). Certain relationships among moderate alcohol consumers were not statistically significant, likely due to the limited sample size of this category [33].

### 4.2. Association Between Serum Lycopene and IGF Levels

Second, lycopene supplementation was associated with lower IGF-I levels than those in the control group. However, this effect was largely driven by a single study, and sensitivity analyses rendered a non-significant association. In contrast, no significant differences were found in IGF-I levels when comparing individuals with high and low dietary lycopene intake, suggesting that the effect of lycopene on IGF-I may be more pronounced in supplementation settings than in habitual diets.

Similarly, no significant differences were observed in IGF-II, IGFBP-2, or IGFBP-3 levels between the lycopene and control groups, whereas IGFBP-1 levels were modestly elevated in lycopene-supplemented individuals. The finding revealing increased IGFBP-1 levels may indicate lycopene’s influence on glucose metabolism and insulin sensitivity, although the clinical significance of the modest increase remains unclear.

These results are consistent with those reported by Xie et al. (2021), who reported no significant associations between lycopene supplementation and serum IGF-1 levels [18]. However, their subgroup analysis revealed that lycopene significantly reduced IGF-1 levels when administered at a dosage of >15 mg/d for less than 12 weeks to healthy adults and those aged > 60 years. Notably, there was no significant association between lycopene consumption and alterations in IGF-1 levels in patients with cancer.

The absence of a significant correlation between lycopene intake and IGF-1 levels is noteworthy. Among the studies included in this systematic review, only one demonstrated an association between smoking and colon cancer [51]. Kucuk et al. (2001) reported that both the intervention and control groups they examined exhibited reductions in serum IGF-1 and IGFBP-3 levels after three weeks of treatment [66]. Overall, the majority of the evidence, including the findings of Vrieling et al. (2007), Tran et al. (2006), and Signorello et al. (2000), supports the conclusion that lycopene supplementation does not significantly impact serum IGF-1 concentrations [51,55,67].

To properly contextualize these findings, it is essential to compare them with the standard reference ranges. Normal serum IGF-1 levels typically range from 116 to 341 ng/mL in individuals aged 21–25 years and from 72 to 207 ng/mL in those aged 61–65 years [68]. Notably, lycopene supplementation appeared to reduce IGF-1 concentrations by approximately 25 ng/dL in individuals aged ≥ 60 years, a substantial change for a nutraceutical intervention. Interestingly, a similar reduction (~26 ng/dL) was observed in healthy individuals compared to that in cancer patients. Theoretically, cancer patients may benefit from decreased IGF-1 levels, given IGF-1’s role as an anabolic hormone involved in oncogenic signaling pathways. However, the lack of robust and consistent changes precludes the use of lycopene supplementation as a strategy for lowering elevated IGF-1 levels.

Mechanistically, IGF-1 is a paracrine/autocrine peptide hormone belonging to the insulin-related peptide family and exists in three isoforms (IGF-1Ea, IGF-1Eb, and IGF-1Ec). While the liver is the primary source of circulating IGF-1, other tissues, such as bone and muscle, also produce it locally [69]. In circulation, IGF-1 primarily binds to IGFBPs, particularly forming ternary complexes with IGFBP-3 and the acid-labile subunit, which regulate the availability of free bioactive IGF-1 [70]. Thus, IGF-1 activity is modulated not only by its serum concentration but also by IGFBPs and related proteases [71,72].

IGF-1, along with insulin, activates the PI3K/AKT/mTOR pathway, promoting cell growth, proliferation, and survival while inhibiting apoptosis, which are key processes that support cancer cell development. Therefore, elevated serum IGF-1 levels have been linked to an increased cancer risk and have been proposed as complementary biomarkers for cancer susceptibility [73]. Paradoxically, IGF-1 is also critical for skeletal muscle protein synthesis and hypertrophy through its interaction with IGF1R and insulin receptors. Consequently, while reducing IGF-1 levels may be advantageous in the context of cancer prevention, it could be detrimental by exacerbating muscle wasting (cachexia) in cancer patients or impairing muscle growth in healthy individuals [74]. Given this complexity, further research is warranted, particularly on healthy populations seeking musculoskeletal gains, although strength training and adequate protein intake remain the primary drivers of muscle hypertrophy, independent of circulating anabolic hormone levels [75].

### 4.3. Association Between Serum Lycopene and Mortality Risk

Importantly, higher serum lycopene levels were associated with a reduced mortality risk among individuals with metabolic diseases. This suggests a potential protective role for lycopene in this high-risk population. However, this conclusion is heavily influenced by a single large study, and further longitudinal research is required to confirm this effect across diverse populations.

Regarding glycemic outcomes, no significant associations were found between serum lycopene and high HbA1c levels or a history of *diabetes mellitus*. The analysis of high HbA1c levels was limited by substantial heterogeneity and divergent results between studies, indicating uncertainty in the results. Meanwhile, the consistently null findings regarding the history of diabetes suggest that lycopene may have little to no direct impact on diabetes incidence.

Finally, although the comparison of serum lycopene levels between patients with diabetes and controls was incomplete in the available data, the initial trends suggested there are lower lycopene levels among individuals with diabetes, in line with prior observational studies linking oxidative stress and micronutrient deficiencies to metabolic disease progression.

The inadequate intake of lycopene among certain individuals may largely stem from diverse dietary habits influenced by cultural practices. Data from the Malaysian Adult Nutrition Survey (MANS) revealed that staple foods for the surveyed group included white rice, marine fish, green leafy vegetables, bread, biscuits, and traditional dishes [76]. In contrast, lycopene, a red pigment mainly found in fruits and vegetables like tomatoes, pink guava, and grapefruits, as well as tomato-based products such as spaghetti, pizza, tomato juice, tomato sauce, and minestrone, is less commonly consumed.

Leh et al. (2021) reported that higher lycopene consumption may offer protective effects against elevated glycemic markers [26]. Previous studies have supported the beneficial effects of dietary lycopene on blood glucose control, glucose tolerance, and HbA1c levels [61,77,78]. However, the findings from epidemiological studies on the role of lycopene in type 2 *Diabetes Mellitus* (T2DM) have been inconsistent. For instance, a double-blind, placebo-controlled trial showed that daily intake of 10 mg of lycopene for two months could help prevent long-term complications in T2DM patients by boosting total antioxidant capacity, reducing malondialdehyde-modified LDL (MDA-LDL) formation, and increasing serum immunoglobulin M1 levels [79]. Another study found that short-term tomato juice supplementation (500 mL/day) lowered LDL oxidation in T2DM patients [80]. Conversely, large cohort studies from Finland [59] and the United States [57] did not observe any significant association between dietary lycopene intake and incidence of T2DM. The possible reasons for these discrepancies include differences in disease stage among patients and limitations inherent to self-reported food frequency questionnaires, which are prone to recall bias.

Evidence suggests that hyperglycemia, hyperinsulinemia, and insulin resistance lead to excessive ROS production, contributing to oxidative stress, which in turn impairs insulin action and glucose metabolism in peripheral tissues, promotes beta-cell dysfunction, and accelerates the progression of diabetes and the development of associated complications [81]. Lycopene, as a potent antioxidant, helps maintain redox balance by scavenging free radicals, thus protecting vital biomolecules like lipids, proteins, and DNA. Owing to its open-chain unsaturated structure, lycopene is particularly reactive with oxygen, making it effective in preventing oxidative degradation [82]. Additionally, it acts as a chain-breaking antioxidant by neutralizing peroxyl radicals during lipid peroxidation [83]. Moreover, lycopene has been shown to inhibit ROS production induced by 7-ketocholesterol in human macrophages, either through direct antioxidant effects or by modulating NADPH oxidase expression [84], which significantly contributes to reducing oxidative stress.

### 4.4. Implications for Future Directions

Collectively, these findings highlight that low serum lycopene levels constitute a potential biomarker for greater metabolic risk, particularly for MAFLD and mortality, among metabolically compromised individuals. However, evidence for the impact of lycopene on IGF signaling and glycemic control is less consistent. Differences in study designs, populations, lycopene measurement methods, and outcome definitions likely contributed to the heterogeneity observed and some conflicting results.

Given the antioxidant properties of lycopene, these findings support further exploration of lycopene supplementation or dietary strategies as potential adjuncts in the management and prevention of metabolic diseases. Nonetheless, in the future, researchers should prioritize the performance of high-quality randomized controlled trials and longitudinal studies with larger, more diverse populations to validate these associations and determine causality, particularly regarding liver health, mortality outcomes, and glycemic regulation.

### 4.5. Strengths and Limitations

A major strength of this study is the comprehensive inclusion of a wide range of study designs and populations, providing a broad perspective on the potential role of lycopene in metabolic health (maximizing the amount of data analyzed to better explore and understand the possible associations despite the inherent variability). However, the limitations include the relatively small number of studies for some outcomes; the substantial heterogeneity observed for some key outcomes, such as IGF-II levels and HbA1c; the fact that all the studies included were written in English, meaning studies published in other languages were not considered; and the observational nature of many of the included studies, which precludes causal inference. Additionally, the heavy influence of single studies on some pooled estimates warrants cautious interpretation. Follow-up values for prospective studies were not uniformly reported across studies and are therefore not reported herein.

Another important limitation is the variability in the definitions of key metabolic outcomes across the included studies’ definitions. For example, while most studies defined obesity using the standard BMI threshold of ≥30 kg/m^2^, some applied region-specific cutoffs, such as ≥25 kg/m^2^ for Asian populations. Similarly, hyperglycemia was variably defined using different diagnostic criteria, including HbA1c levels and fasting plasma glucose levels, with inconsistent thresholds. This definitional heterogeneity may contribute to measurement bias and affect the comparability of effect estimates between studies. We considered their impact on the interpretation of the findings; however, the limited number of eligible studies precluded the performance of formal subgroup analysis to fully assess this variability.

Several factors may underlie the observed inconsistencies and heterogeneity across the studies included in this meta-analysis. One key source of variability is the genetic background of the study population, which can influence lycopene absorption, metabolism, and antioxidant activity. For example, polymorphisms in genes involved in lipid metabolism may affect the transport and conversion of lycopene, potentially modifying its effects.

In addition to genetic variability, lifestyle and environmental factors likely contributed to the heterogeneity of the findings. Differences in dietary patterns, including the overall intake of fruits and vegetables, fat content (which affects lycopene absorption), and cooking methods (which impact bioavailability), may influence serum lycopene levels and their relationship with metabolic outcomes. Moreover, other lifestyle variables, such as physical activity, smoking status, alcohol consumption, and supplement use, vary substantially across populations and may confound or modify associations.

Regional differences in healthcare access, nutritional education, and screening practices for metabolic diseases could also shape the apparent strength of the associations reported in primary studies. Finally, variations in study design (e.g., cross-sectional vs. longitudinal), measurement tools, and statistical adjustments for confounders may have further contributed to the inconsistent results.

Such heterogeneity complicates the pooling of results and may reduce the precision of estimated effects. Additionally, many of the included studies exhibited a moderate-to-serious risk of bias due to factors such as inadequate blinding, small sample sizes, and incomplete outcome reporting. These biases could influence the effect estimates and limit confidence in our findings. Together, the presence of heterogeneity and a risk of bias may affect the interpretation and generalizability of our results. Recognizing these limitations is important for providing a nuanced and transparent synthesis of the current evidence and highlights the need for more rigorous and well-powered studies to validate these associations.

Taken together, these population-specific and methodological differences highlight the importance of conducting future studies that stratify by or adjust for genetic and lifestyle factors to more precisely estimate the relationship between lycopene and metabolic health outcomes.

## 5. Conclusions

This combined systematic review and meta-analysis provides evidence supporting an inverse association between serum lycopene levels and MAFLD risk. However, the role of lycopene in glycemic control, obesity, and diabetes remains inconclusive based on the current data. While these findings suggest lycopene plays a protective role in liver-related metabolic health, inconsistencies across studies highlight the need for further research. Moreover, the substantial impact of individual studies on certain pooled estimates necessitates cautious interpretation of the results. Future research should prioritize high-quality randomized controlled trials and longitudinal studies with larger sample sizes to verify the associations identified in this meta-analysis. Additionally, investigating the biological mechanisms linking lycopene to metabolic outcomes will help build a more robust evidence base for future clinical applications of lycopene. Until then, dietary intake of lycopene-rich foods may be cautiously encouraged as part of a balanced diet for general metabolic health.

## Figures and Tables

**Table 1:** Summary of the included studies.

Study ID	Setting (Country)	Type of Study	Type of Metabolic Disease	Sample Size	
				Study Group	Comparision Group
Chai et al., 2024 [33]	US	Cross-sectional	MAFLD	1148	2808
Lin et al., 2024 [34]	US	Cross-sectional	MAFLD	3040	
Yu et al., 2024 [35]	US	Cross-sectional	MAFLD	2925	2173
Yu et al., 2023 [36]	China	Cross-sectional	Obesity	10,192	15,676
Zhang et al., 2023 [37]	US	Cross-sectional	MAFLD	1476	1246
Leh et al., 2021 [38]	Malaysia	Case–control	Hyperglycemia	87	122
Biernacka et al., 2019 [39]	UK	Randomized clinical trial	IGF Decline	Supplement: 41 Dietary Advice: 42	37
Christensen et al., 2019 [40]	US	Cross-sectional	MAFLD	30,295,373	61,456,846
Sanjeevi et al., 2019 [41]	US	Secondary analysis of randomized clinical trial	Hyperglycemia	136	
Cao et al., 2015 [42]	China	Cross-sectional	MAFLD	1486	1449
Sugiura et al., 2015 [43]	Japan	Follow-up study on Mikkabi cohort study	Hyperglycemia	864	
Li et al., 2013 [44]	China	Cross-sectional	Metabolic Syndrome	221	323
Suzuki et al., 2011 [45]	Japan	Cross-sectional	Metabolic Syndrome	117	814
Coyne et al., 2009 [46]	Australia	Cross-sectional	Metabolic Syndrome	424	1112
Markovits et al., 2009 [47]	Israel	Clinical Trial	Obesity	8	8
Suzuki et al., 2009 [48]	Japan	Prospective cohort	IGF Decline	924	
Sugiura et al., 2008 [49]	Japan	Cross-sectional	Metabolic Syndrome	59	899
Voskuil et al., 2008 [50]	Netherlands	Randomized clinical trial	IGF Decline	60	
Vrieling et al., 2007 [51]	Netherlands	Randomized clinical trial	IGF Decline	71	
Walfisch et al., 2007 [52]	Israel	Randomized clinical trial	IGF Decline	30	26
Kimmons et al., 2006 [53]	US	Cross-sectional	Obesity	16,191	
Suzuki et al., 2007 [54]	Japan	Cross-sectional	Obesity	174	416
Tran et al., 2006 [55]	Canada	Cross-sectional	IGF Decline	1542	
Wang et al., 2006 [56]	US	Prospective case–control design	Hyperglycemia	470	470
Wang et al., 2005 [57]	US	Prospective cohort	Hyperglycemia	35,783	
Coyne et al., 2005 [58]	Australia	Cross-sectional	Hyperglycemia	1597	
Montonen et al., 2004 [59]	Finland	Prospective cohort	Hyperglycemia	383	3921
Ford et al., 2003 [60]	US	Cross-sectional	Metabolic Syndrome	8808	
Suzuki et al., 2002 [61]	Japan	Cross-sectional	Hyperglycemia	High HbA1c: 151 DM: 133	Group 1: 302 Group 2: 266

## Data Availability

All data generated or analyzed during this study are included in this published article [and its Supplementary Information files].

## Supplementary Materials

The following supporting information can be downloaded at https://www.mdpi.com/article/10.3390/life15060944/s1. Figure S1: Sensitivity analysis of association between serum lycopene and MAFLD Risk. Figure S2: Sensitivity analysis of mean difference in IGF-I levels: lycopene vs. control. Figure S3: Sensitivity analysis of mean difference in IGF-I levels: highest vs. lowest lycopene quantiles. Figure S4: Sensitivity analysis of mean difference in IGF-II Levels: lycopene vs. control. Figure S5: Sensitivity analysis of mean difference in IGFBP-1 Levels: lycopene vs. control. Figure S6: Sensitivity analysis of mean difference IGFBP-2 Levels: lycopene vs. control. Figure S7: Sensitivity analysis of mean difference in IGFBP-3 Levels: lycopene vs. control. Figure S8: Sensitivity analysis of association between serum lycopene and history of diabetes mellitus. Figure S9: Sensitivity analysis of mean difference in serum lycopene levels: diabetic vs. control. Figure S10: Sensitivity analysis of mean difference in serum lycopene levels: normal weight vs. obesity. Figure S11: Funnel plots showing asymmetry and symmetry of (a) association between serum lycopene and MAFLD Risk (b) IGF-I levels: lycopene vs. control and (c) IGF-II Levels: lycopene vs. control. Figure S12: Funnel plots showing asymmetry and symmetry of the outcomes: (a) IGFBP-1 Levels: lycopene vs. control (b) IGFBP-2 Levels: lycopene vs. control and (c) IGFBP-3 Levels: lycopene vs. control. Figure S13: Funnel plots showing asymmetry and symmetry of the outcomes: (a) Association between serum lycopene and history of diabetes mellitus and (b) mean difference in serum lycopene levels: diabetic vs. control. Table S1: Details of search strategy. Table S2: Details of baseline characteristics of the included patients. File S1: Terms for the research strategy.

## Abbreviations

The following abbreviations are used in this manuscript:

BMI	Body mass index
CI	Confidence intervals
HR	Hazard ratio
IGF	Insulin-like growth factor
IGFBPs	Insulin-growth-factor-binding proteins
MAFLD	Metabolic fatty liver disease
MD	Mean difference
MDA-LDL	Malondialdehyde-modified LDL
NAFLD	Nonalcoholic fatty liver disease
OR	Odds ratio
RCT	Randomized controlled trial
ROS	Reactive oxygen species
T2DM	Type 2 *Diabetes Mellitus*
