# Supplementary material for: Association Between Lycopene and Metabolic Disease Risk and Mortality: Systematic Review and Meta-Analysis
Source: Life (Basel). 2025 Jun 12;15(6):944. doi: 10.3390/life15060944 (PMC12194687; doi:10.3390/life15060944)
Supplement: Supplementary file 1 [file life-15-00944-s001.zip › Supplementary Figure S12.pdf]

Supplementary Figure S12

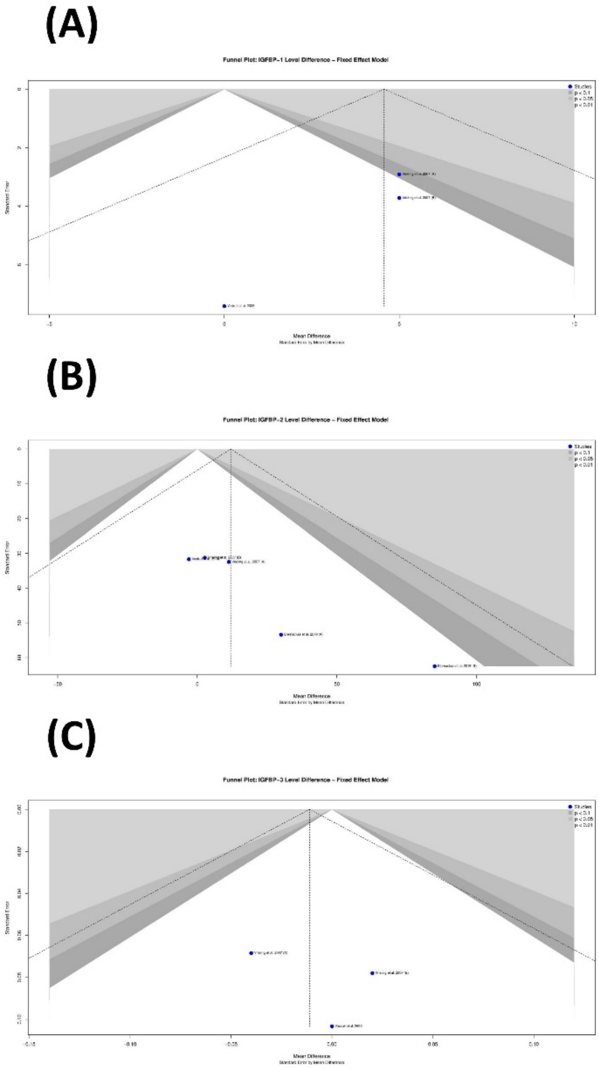

Supplementary Figure S12. Funnel plots showing asymmetry and symmetry of the outcomes: (a) *IGFBP-1 Levels: Lycopene vs Control* (b) IGFBP-2 Levels: Lycopene vs Control and (c) IGFBP-3 Levels: Lycopene vs Control
