# Supplementary material for: Association Between Lycopene and Metabolic Disease Risk and Mortality: Systematic Review and Meta-Analysis
Source: Life (Basel). 2025 Jun 12;15(6):944. doi: 10.3390/life15060944 (PMC12194687; doi:10.3390/life15060944)
Supplement: Supplementary file 1 [file life-15-00944-s001.zip › Supplementary Figure S3.pdf]

Supplementary Figure S3

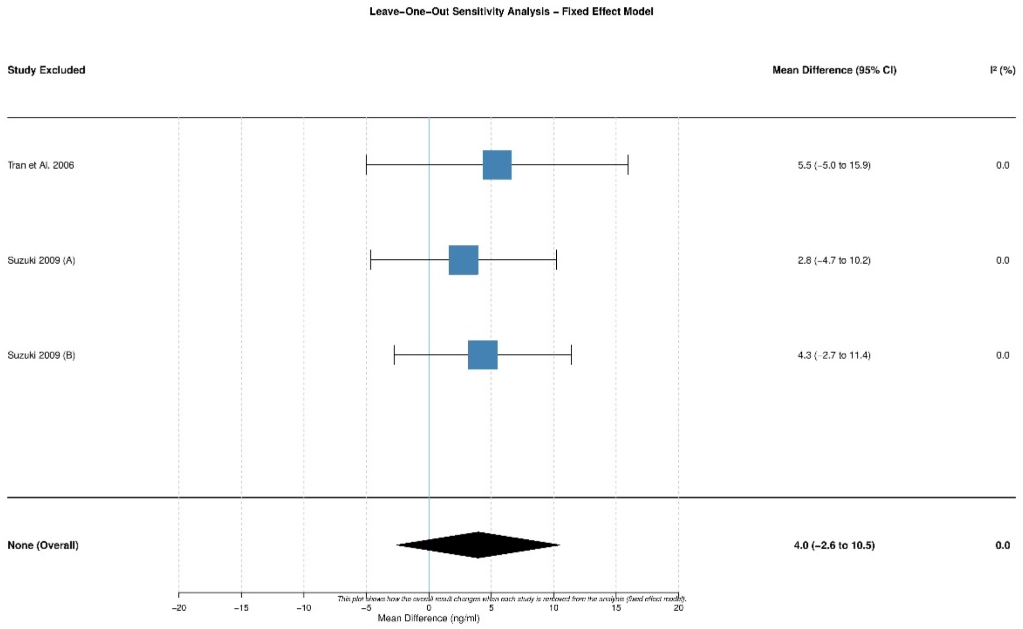

Supplementary Figure S3. Sensitivity analysis of mean difference in IGF-I Levels: Highest vs Lowest Lycopene Quantiles
