# Supplementary material for: Association Between Lycopene and Metabolic Disease Risk and Mortality: Systematic Review and Meta-Analysis
Source: Life (Basel). 2025 Jun 12;15(6):944. doi: 10.3390/life15060944 (PMC12194687; doi:10.3390/life15060944)
Supplement: Supplementary file 1 [file life-15-00944-s001.zip › Supplementary File S1.pdf]

Supplementary File S1: Terms for the research strategy:

(Lycopene OR Carotenoids OR Lycopene, (13-cis)-isomer OR Lycopene, (7-cis,7'-cis,9-cis,9'-cis)-isomer OR Prolycopene OR Pro-Lycopene OR Pro Lycopene OR Lycopene, (cis)-isomer OR LYC-O-MATO OR LYCOMATO OR LYC O MATO OR All-trans-Lycopene OR All trans Lycopene OR lycopene beta-cyclase OR lycopene cyclase OR lycopene epsilon-cyclase) AND (Metabolic Syndrome OR Insulin Resistance OR Obesity OR Diabetes OR Abdominal Obesity-Metabolic Syndrome OR Non-alcoholic fatty liver OR Metabolic associated fatty liver)
