# Supplementary material for: Association Between Lycopene and Metabolic Disease Risk and Mortality: Systematic Review and Meta-Analysis
Source: Life (Basel). 2025 Jun 12;15(6):944. doi: 10.3390/life15060944 (PMC12194687; doi:10.3390/life15060944)
Supplement: Supplementary file 1 [file life-15-00944-s001.zip › Supplementary Table S2.pdf]

Supplementary S2. Characteristics of the studies included.

	Age mean ± SD		Male sex (%)		Education (%)		Physical activity (%)		Smoking Status (%)		Alcohol Consumption (%)		BMI mean ± SD		Total Cholesterol mean ± SD		Triglyceride mean ± SD	
Study ID	Cases	Controls	Cases	Controls	Cases	Controls	Cases	Controls	Cases	Controls	Cases	Controls	Cases	Controls	Cases	Controls	Cases	Controls
Chai et al, 2024 [33]	51.4±0.7	46.7±0.7	61.80%	45.70%	Below high school (10.5%) High school (63.9%) Above high school (25.6%)	Below high school (10.3%) High school (56.6%) Above high school (33.2%)	NR	NR	Never (51.1%) Current (16.1%) Former (32.8%)	Never (58.8%) Current (18.5%) Former (22.6%)	Never/rarely (35.6%) Occasionally (23.2%) Sometimes (29.3%) Frequently (11.9%)	Never/rarely (30.9%) Occasionally (21.3%) Sometimes (32.9%) Frequently (14.9%)	34.9±0.5	27.6±0.3	NR	NR	150±9.3	99.4±2.2
Lin et al, 2024 [34]	47.02±0.43		56.92%		Below high school (28.24%) High school (37.77%) Above high school		NR		Never (42.22%) Current (24.49%) Former (33.29%)		19.23%		30.71±0.22		5.52±0.04		2.26±0.06	

					(33.99%)													
Yu et al, 2024 [35]	18-29y (11.6% 30-49y (33%) 50-69y (40.6% >70y (14.8%)	18-29y (26.4%) 30-49y (36.6%) 50-69y (26.9%) >70y (10.1%)	1482 (53.3%)	973 (42.9%)	Below high school 477 (10%) High school 708 (28.7%) Above high school 1740 (61.3%)	Below high school 297 (7.5%) High school 474 (23.5%) Above high school 1402 (68.9%)	Low 761 (22%) Moderate 1139 (38.7%) High 1025 (39.3%)	Low 427 (14.3%) Moderate 889 (43.7%) High 857 (42%)	Never 1635 (55.7%) Current 457 (15.2%) Former 833 (29.1%)	Never 1317 (62.6%) Current 436 (16.4%) Former 420 (21%)	Non-drinkers 2137 (69.1%) Drinkers 690 (25.6%) Heavy Drinkers 98 (5.2%)	Non-drinkers 1517 (65.4%) Drinkers 611 (32.6%) Heavy Drinkers 45 (2%)	NR	NR	NR	NR	NR	NR
Yu et al, 2023 [36]	20- 39y (28.4%) 40-59y (36.5%) >60y (35.1%)	20-39y (33%) 40-59y (32.1%) >60y (34.9%)	44.30 %	51.50 %	Below high school (24%) High school (24.4%) Above high school	Below high school (22%) High school (22.1%) Above high school	0: (30.7%) 0.01 <20: (24.3%) 20 to <68: (22%) >68:	0: (23.6%) 0.01 <20: (24.2%) 20 to <68: (26.6%)	Never 5683 (55.8%) Current 1787 (17.5%) Former	Never 8685 (55.4%) Current 3294 (21%) Former	Yes 5548 (68.9%) No 2507 (31.1%)	Yes 9411 (74.3%) No 3263 (25.7%)	NR	NR	NR	NR	NR	NR

					(51.6%)	(55.9%)	(23%)	>68: (25.6%)	er 2717 (26.7%)	3690 (23.6%)								
Zhang et al, 2023 [37]	49.82±34.4	42.53±26.3	57.12%	46.89%	Below high school (9.51%)<br>High school (29.71%)<br>Above high school (60.78%)	Below high school (7.97%)<br>High school (25.29%)<br>Above high school (66.74%)	Low (16.65%)<br>Moderate (58.24%)<br>High (25.11%)	Low (9.19%)<br>Moderate (66.62%)<br>High (24.19%)	Never (56.34%)<br>Current (16.59%)<br>Former (27.07%)	Never (61.29%)<br>Current (18.5%)<br>Former (20.20%)	NR	NR	Underweight or Normal (<25): 5.67%<br>Overweight (25to <30): 32.01%<br>Obese (30 or greater): 62.33%	Underweight or Normal (<25): 52.94%<br>Overweight (25to <30): 29.32%<br>Obese (30 or greater): 17.75%	NR	NR	1.57±3.3	0.95±1.2

Leh et al, 2021 [38]	56.3±4.6		NR	NR	NR	NR	NR	NR	NR	NR	NR	NR	28.5 ±5.3	27.9±3.3	4.6±1.1	4.5±0.8	1.8± 1.0	1.6±0.6
Biernacka et al, 2019 [39]	Supplement: 65±6.2 Dietary Advice: 64±4.7	64±4.9	83 (100%)	37 (100%)	NR	NR	NR	NR	NR	NR	NR	NR	Supplement: 26±2.7 Dietary Advice: 27±3.7	28±4.2	NR	NR	NR	NR
Christensen et al, 2019 [40]	20-39y (21.1%) 40-59y (38.4%) 60-79y (35.2%) >80y (5.3%)	20-39y (35.8%) 40-59y (35.5%) 60-79y (22.4%) >80y (6.3%)	16,577,926 (54.7%)	26,036,711 (42.4%)	Below high school 6,847,959 (22.6%) High school 17,238,418 (56.9%) Above high school	Below high school 9,969,649 (16.2%) High school 31,596,068 (51.4%) Above high school	NR	NR	Never 17,112,945 (56.5%) Current 3,937,101 (13%) Former 9,242,180 (30.5	Never 39,936,202 (65%) Current 8,772,599 (14.3%) Former 12,734,901 (20.7	NR	NR	Underweight (<18.5): 11,483 (0%) Normal (18.5-24.9): 1,067,42	Underweight (<18.5): 1,267,523 (2.1%) Normal (18.5-24.9): 23,267,000 (37.9%) Overweight (25-29.9): 24,039,	194.76 ±1.33	196.21±1	175.63±4.18	107.73±2.15

				6,208,995 (20.5%)	19,875,667 (32.3%)			%)	%)			9 (3.5%) Overweight (25-29.9): 6,865,896 (22.7%) Obese (>30): 22,350,563 (73.8%)	650 (39.1%) Obese (>30): 12,882,671 (21%)				
Sanjeevi et al, 2019 [41]	12.7±2.6		NR	NR		NR		NR		NR		21.3±4.2		NR		NR	
Cao et al, 2015	60.8±5.7	60.6±6.1	929 (31.7%)	NR	NR	33.6±1.2	34.3±5.7	174 (11.7	158 (10.9	NR	NR	25±3	22.1±2.6	NR	NR	NR	NR

[42]									%)	%)								
Sugiura et al, 2015 [43]	All baseline characteristics of this study weren't reported																	
Li et al, 2013 [44]	54.2±5.73	53.3±5.83	81 (36.7%)	130 (40.2%)	NR	NR	NR	NR	NR	NR	NR	NR	26.9±3.21	24.1±4.13	4.94±1.33	5±1.04	2.13±1.28	1.18±0.82
Suzuki et al, 2011 [45]	Males: 60.7±7.8 Females: 62.7±5.4	Males: 59.4±7.8 Females:57.6±8.2	71 (60.7%)	247 (30.3%)	NR	NR	NR	NR	Never (Males): 13 (18.3%) Current 26 (36.6%) Former 32 (45.1%) Never (Females): 34 (73.9	Never (Males): 44 (17.8%) Current 88 (35.6%) Former 115 (46.6%) Never (Females): 434	Never (Males): 17 (24.3%) Current 50 (71.4%) Former 3 (4.3%) Never (Females): 36 (78.3%) Current 8 (17.4%) Former 2 (4.4%)	Never (Males): 70 (28.3%) Current 162 (65.6%) Former 15 (6.1%) Never (Females): 420 (74.2%) Current 131 (23.1%) Former 15 (2.7%)	Males: 27.1±3.3 Females: 29.3±3.8	Males: 23.3±2.8 Females:23.3±3.2	Males: 208.2±29.2 Females:212.8±37	Males: 206±32.5 Females: 220.2±35.1	Males: 162.4(103-218) Females: 145.4 (102-195.5)	Males: 97.7 (70-129) Females: 87.1 (65-115)

									%) Current 5 (10.9%) Former 7 (15.2%)	(76.5%) Current 73 (12.9%) Former 60 (10.6%)								
Coyne et al, 2009 [46]	25-34y: 19 (11%) 35-44y: 48 (14.6%) 45-54y: 114 (29.4%) 55-64y: 120 (38.4%) 65-74y: 88 (38%) >75y: 35 (36.2%)	25-34y: 161 (89%) 35-44y: 275 (85.4%) 45-54y: 270 (70.6%) 55-64y: 199 (61.7%) 65-	195 (26.7%)	451 (73.3%)	High school or less: 191 (27.5%) Trade certificate, bachelor`s degree: 214 (23%) Post-graduate qualification	High school or less: 415 (72.5%) Trade certificate, bachelor`s degree: 598 (77%) Post-graduate qualification	Sufficiently Active: 179 (22%) Insufficiently Active: 134 (22.2%) Sedentary: 111 (33.1%)	Sufficiently Active: 542 (78%) Insufficiently Active: 380 (77.8%) Sedentary: 185 (66.9%)	Never 218 (20.8%) Current 59 (25.1%) Former 142 (29.8%)	Never 660 (79.2%) Current 147 (74.9%) Former 289 (70.2%)	None: 105 (28%) <60 standard drinks/month: 265 (22.1%) >60 standard drinks/month: 54 (27.8%)	None: 233 (72%) <60 standard drinks/month: 761 (77.9%) >60 standard drinks/month: 118 (72.2%)	Normal (<25): 27 (3.2%) Overweight (>25 to <30): 189 (28.3%) Obese (>30): 208 (57.2	Normal (<25): 584 (96.8%) Overweight (>25 to <30): 379 (71.7%) Obese (>30): 149 (42.8%)	NR	NR	2.54±0.14	1.13±0.03

		74y: 145 (62%) >75y: 62 (63.8%)			: 19 (15.8 %)	: 95 (84.2 %)							%)					
Markovits et al, 2009 [47]	47±6	Age-matched	4 (50%)	4 (50%)	NR	NR	NR	NR	NR	NR	NR	NR	37.5 ± 7.1	21.6 ± 1.7	NR	NR	199.4 ± 122.8	98 ± 42.7
Suzuki et al, 2009 [48]	Males: 64.8±7.5 Females: 62.1±8.6		578 (63%)		NR		NR		Males: Never 122 (21.1%) Current 433 (74.9%) Former 23 (4%) Females: Never 270 (78%) Current 73 (21.1%) Former 3 (0.9%)		Current (Males): 271 (46.9%) Former Drinker 182 (31.5%) Non-drinker 125 (21.6%) Current (Females): 9 (2.6%) Former drinker 7 (2%) Non-drinker 330 (95.4%)		Males: 22.2±2.7 Females: 22.9±3.0		Males: 191.9±36.4 Females: 212.0±38.7		NR	
Sugiura et al, 2008 [49]	NR	NR	NR	NR	NR	NR	NR	NR	12	100	NR	NR	NR	NR	NR	NR	NR	NR

Voskuil et al, 2008 [50]	NR		0 (0%)		NR		NR		NR		NR		NR		NR		NR	
Vrieling et al, 2007 [51]	NR		40 (56%)		NR		NR		NR		NR		NR		NR		NR	
Walfisch et al, 2007 [52]	69±2	68±2	18 (60%)	17 (65%)	NR		NR		Yes :3 No: 23 Unknown: 3 Former: 1	Yes :1 No: 21 Unknown: 2 Former: 2	NR		26.7 ±1.4	28±0.9	NR		NR	
Kimmons et al, 2006 [53]	NR	NR	7808 (48.2%)		NR	NR	NR	NR	NR	NR	NR	NR	NR	NR	NR	NR	NR	NR
Suzuki et al, 2007 [54]	Males: 60+10.6 Females: 58.8+10.1		192 (32.5%)		NR	NR	Little (Males): 40 (72.7%) 1-2h/week or more (Males	Little (Males): 87 (63.5%) 1-2h/week or more (Males	Never (Males): 13 (23.6%) Current 19 (34.5	Never (Males): 26 (19%) Current 53 (38.7%)	Current (Males): 24 (43.6%) Former Drinker 2 (3.6%) Non-drinker 29	Current (Males): 74 (54%) Former Drinker 10 (7.3%) Non-drinker 53 (38.7%)	Males: 23.6+2.8 Females: 23.4+3		Males: 209.8+31.4 Females: 218.8+34.5		Males: 97.3 (70-126.3) Females: 80 (59-104)	

): 15 (27.3%) Little (Females); 76 (63.9%) 1-2 h/week or more (Females): 42 (35.3%)	): 50 (36.5%) Little (Females): 179 (64.2%) 1-2 h/week or more (Females): 101 (36.2%)	%) Former 23 (41.8%) Never (Females): 98 (82.4%) Current 8 (6.7%) Former 12 (10.1%)	Former 58 (42.3%) Never (Females): 221 (79.2%) Current 31 (11.1%) Former 28 (10%)	(52.7%) Current (Females): 19 (16%) Former drinker 2 (1.7%) Non-drinker 97 (81.5%)	Current (Females): 44 (15.8%) Former drinker 9 (3.2%) Non-drinker 227 (81.4%)

Tran et al, 2006 [55]	54.1±9.4	0 (0%)	Primary or less: 16.3% Secondary: 32.6% College: 19.6% University: 31.5%	26.3±22.8	Never (52.1%) Current (14.1%) Former (33.8%)	0 drinks/wk: 12.3% >0 to <1 36.1% >1 to <6: 36.9% >6: 14.7%	26.1±4.7	NR	NR

Wang et al, 2006 [56]	55.7±7.0	55.7±7.0	470 (100%)	470 (100%)	NR	NR	Rarely/Never: 49.7% <1 time/week: 23.0% 1-3 times/week: 21.3% >4 times/week: 6.0%	Rarely/Never: 53.3% <1 time/week: 19.2% 1-3 times/week: 32.1% >4 times/week: 13.4%	Never (53.2%) Current (13.8%) Former (33.0%)	Never (54.1%) Current (10.4%) Former (35.5%)	Rarely/Never: 60.8% 1-3 drinks/month: 14.7% 1-6 drinks/week: 21.3% >1 drink/day: 3.2%	Rarely/Never: 41.4% 1-3 drinks/month: 13.2% 1-6 drinks/week: 34.3% >1 drink/day: 11.1%	31.8±6.2	25.6±4.9	NR	NR	NR	NR
Wang et al, 2005 [57]	54.5±7.0		0 (0%)		NR		NR		NR		NR		NR		NR		NR	
Coyne et al, 2005 [58]	Impaired Glucose Tolerance 25-34y: 12 (54.8%) 35-44y: 39 (11.6%) 45-54y: 68	25-34y: 170 (94.5%) 35-44y: 286 (86.7%) 45-54y: 298 (76.4%	Impaired Glucose Tolerance: 132 (15.9%) T2DM: 63 (6.9%	484 (77.2%)	Impaired Glucose Tolerance: High school or less: 145 (20.1	High school or less: 418 (71.4%), Trade certificate, bachelor`s	Impaired Glucose Tolerance: Sufficiently Active: 135 (14.8%)	Sufficiently Active: 560 (79.8%) Insufficiently Active: 374 (74.7%)	Impaired Glucose Tolerance: Never 185 (17.3%) Curre	Never 655 (76.4%) Current 168 (84.4%) Former 306	Impaired Glucose Tolerance: None: 84 (19.7%) <60 standard drink/month: 195 (15.5%) >60 standard	None: 233 (70.1%) <60 standard drink/month: 789 (78.8%) >60 standard drinks/month: 123	Impaired Glucose Tolerance: Normal (<25): 84 (11.0	Normal (<25): 519 (85.6%) Overweight (>25 to <30): 417 (77.7%) Obese (>30): 206	Impaired Glucose Tolerance: <5.5 mm0l/L: 130 (14.3%) >5.5 mmol/	<5.5 mm0l/L: 555 (81.1%) >5.5 mmol/L: 590 (71.3%)	Impaired Glucose Tolerance: <2.0 mmol/L: 208 (14.0	<2 mmol/L:955 (82.1%) >2 mmol/L: 190 (54.2%)

(16.6%) 55-64y: 83 (24.7%) 65-74y: 81 (33.0%) >75y: 37 (33.8%) T2DM: 25-34y: 0 (0%) 35-44y: 7 (1.7%) 45-54y: 29 (6.9%) 55-64y: 31 (10.4%) 65-74y: 39 (16.5%) >75y: 26 (26.6%)	) 55-64y: 220 (64.9%) 65-74y: 125 (50.4%) >75y: 46 (39.6%)	)	%), Trade certificate, bachelor`s degree: 155 (15.1%) Post-graduate qualification: 20 (16.1%) T2DM: High school or less: 63 (8.5%), Trade certificate, bachelor`s	degree: 631 (78.3%) Post-graduate qualification: 92 (81.1%)	Insufficiently Active: 112 (17.9%) Sedentary: 73 (21.2%) T2DM: Sufficiently Active: 51 (5.4%) Insufficiently Active: 46 (7.5%) Sedentary: 35 (9.7%)	Sedentary: 206 (69.1%)	nt 27 (9.6%) Former 104 (20.2%) T2DM: Never 65 (6.3%) Current 20 (5.9%) Former 44 (8.1%)	(71.7%)	drinks/month: 41 (20.1%) T2DM: None: 41 (10.2%) <60 standard drink/month: 74 (5.7%) >60 standard drinks/month: 17 (7.5%)	(72.4%)	%) Overweight (>25 to <30): 124 (17.0%) Obese (>30): 110 (27.5%) T2DM: Normal (<25): 24 (3.0%) Overweight (>25 to <30): 40	(55.9%)	L: 190 (19.5%) T2DM: <5.5 mm0l/L: 44 (4.6%) >5.5 mmol/L: 88 (9.1%)	%) >2 mmol/L: 112 (27.7%) T2DM: <2 mmol/L: 59 (3.9%) >2 mmol/L: 73 (18.0%)

					degree: 66 (6.5%) Post-graduate qualification: 3 (1.9%)								(5.2%) Obese (>30): 67 (16.6%)					
Montonen et al, 2004 [59]	53.7±8.0	51.7±7.6	47%	54%	NR	NR	NR	NR	Current: 28%	Current: 33%	NR	NR	29.4±4.5	26.2±3.8	NR	NR	NR	NR
Ford et al, 2003 [60]	54.3±0.7	41.0±0.4	48.20%	50.10%	NR	NR	Moderate or Vigorously Active: 33.1%	Moderate or Vigorously Active: 43.4%	NR	NR	NR	NR	31.79±0.21	25.28±0.08	5.64±0.05	5.24±0.02	2.60±0.08	1.25±0.02
Suzuki et al, 2002 [61]	High HbA1c:63.6±9.9 DM: 63.8±9.7	Group 1 : 63.1±9.4 Group 2: 63.7±9.61	High HbA1c: 81 (53.6%) DM: 73 (54.9%)	Group 1: 162 (53.6%) Group 2: 146 (54.9	NR	NR	High HbA1c: None: 100 (66.2%), 1-2 Hours/week: 27	Group 1 : None: 207 (68.5%), 1-2 Hours/week: 43	High HbA1c: Never 74 (49.0%) Current 42	Group 1: Never 170 (56.3%) Current 81 (26.8	High HbA1c: Never 79 (52.3%) Current 64 (42.4%) Former 7 (4.6%)	Group 1: Never 206 (68.2%) Current 87 (28.8%) Former 7 (2.3%) Group 2: Never 183	High HbA1c: 24.8±3.6 DM: 24.5±3.3	Group 1: 23.5±2.9 Group 2: 23.2±2.8	High HbA1c: 220.2±38.1 DM: 209.0±31.6	Group 1: 215.5±37.1 Group 2: 210.4±33.9	High HbA1c: 123 (66.6-264.2) DM:	Group 1: 97.5 (54.0-186.3) Group 2: 91.7 (52.0-173.9)

%)	(17.9%), 3-4 hours/ week: 9 (6.0%), 5+ hours/ week: 13 (8.6%) DM: None: 80 (60.2%), 1-2 Hours/ week: 20 (15.0%), 3-4 hours/ week: 14 (10.5%), 5+ hours/ week: 16 (12.0%	(14.2%), 3-4 hours/ week: 25 (8.2%), 5+ hours/ week: 27 (8.9%) DM: None: 179 (67.3%), 1-2 Hours/ week: 35 (13.2%), 3-4 hours/ week: 25 (9.4%), 5+ hours/ week: 25 (9.4%)	(27.8%) Former 35 (23.2%) DM: Never 63 (47.4%) Current 36 (27.1%) Former 34 (25.6%)	%) Former 51 (16.9%) Group 2: Never 142 (53.4%) Current 70 (26.3%) Former 54 (20.3%)	DM: Never 77 (57.9%) Current 39 (29.3%) Former 17 (12.8%)	(68.8%) Current 74 (27.8%) Former 8 (3.0%)		117.9 (64.6-234.4)

)
